# Fabrication and application of biocompatible nanogenerators

**DOI:** 10.1016/j.isci.2021.102274

**Published:** 2021-03-05

**Authors:** Yong-Mei Wang, Qingfeng Zeng, Lilong He, Pei Yin, Yu Sun, Wen Hu, Rusen Yang

**Affiliations:** 1 Academy of Advanced Interdisciplinary Research, School of Advanced Materials and Nanotechnology, Xidian University, Xi'an 710126, China; 2 Joint Laboratory for Intelligent Biofabrication, School of Advanced Materials and Nanotechnology, Xidian University, Xi'an 710126, China; 3 Science and Technology on Thermostructural Composite Materials Laboratory, School of Materials Science and Engineering, Northwestern Polytechnical University, Xi'an 710072, China; 4 MSEA International Institute for Materials Genome, Gu'an 065500, Hebei, China; 5 Xi'an Chuanglian Electronic Component (Group) Co. Ltd., Xi'an 710065, China

**Keywords:** Nanotechnology, Biomaterials, Nanomaterials, Energy Materials

## Abstract

As a new sustainable energy source, ubiquitous mechanical energy has received great attention and was successfully harvested by different types of nanogenerators. Among them, biocompatible nanogenerators are of particular interests due to their potential for biomedical applications. In this review, we provide an overview of the recent achievements in the fabrication and application of biocompatible nanogenerators. The development process and working mechanism of nanogenerators are introduced. Different biocompatible materials for energy harvesting, such as amino acids, peptide, silk protein, and cellulose, are discussed and compared. We then discuss different applications of biocompatible nanogenerators. We conclude with the challenges and potential research directions in this emerging field.

## Graphical abstract

**Figure:**
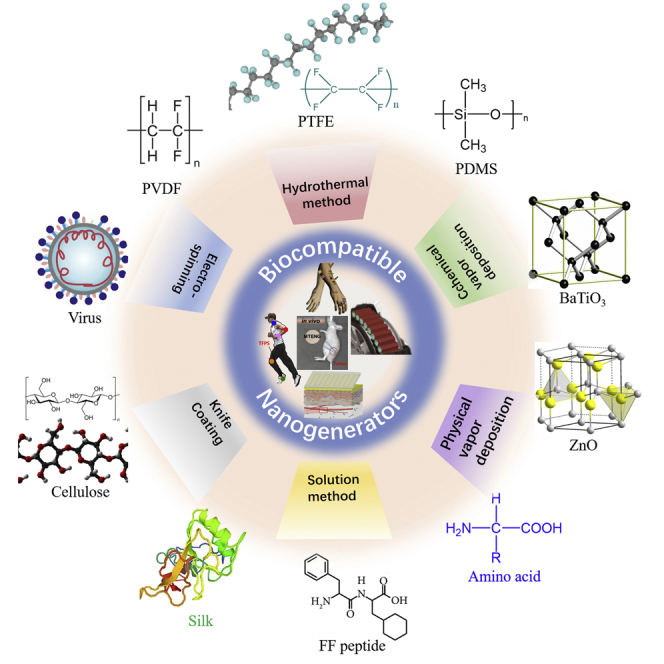

## Introduction

In recent years, emerging technologies have greatly changed our ways of life with devices such as smart watch, smart home, smart phone, and smart car. Long-time and environment-friendly energy supply for these devices is of great importance. Batteries provide a convenient solution, and great efforts have been made to extend their capacity and reduce their impact on the environment. The invention of a piezoelectric nanogenerator (PENG) in 2006 and a triboelectric nanogenerator (TENG) in 2012 uncovered new approaches for power supply and enabled the rapid development of self-powered systems in many fields (Askari et al., 2018a, 2018b, 2019; Chen et al., 2019a; Fan et al., 2012; Wu et al., 2019; Wang and Song, 2006).

A nanogenerator may consist of zinc oxide (ZnO), lead zirconate titanate (PZT), polytetrafluoroethylene (PTFE), and barium titanate (BTO) as energy conversion materials (Chen et al., 2010; Qi et al., 2011; Shin et al., 2014; Tan et al., 2018; Yang et al., 2012c). As the exploration of nanogenerators extended into such fields as human-machine interface, health monitoring, automotive systems, and wearable electronics, biocompatible materials received increasing attention and different biocompatible nanogenerators were fabricated (Askari et al., 2018a; Chen et al., 2019b; He et al., 2018; Hwang et al., 2015; Parida et al., 2019; Song et al., 2019; Sun et al., 2017; Zou et al., 2020). Amino acids with chiral symmetry groups and hierarchical silk, collagen, cellulose and chitin with fibrous structures were explored as piezoelectric materials for biocompatible PENGs (He et al., 2018; Yuan et al., 2019; Li et al., 2020b; Nguyen et al., 2016; Wang et al., 2018). Meanwhile, cellulose, spider silk, inion skin, and other polymers have also been used widely to fabricate a biocompatible TENG (Karan et al., 2018; Liu et al., 2017; Sun et al., 2017; Wang et al., 2017, 2018). The investigation of biocompatible energy conversion materials enabled the development of biocompatible nanogenerators and their applications in health monitoring, biosensing, implantable devices, drug delivery, and tissue engineering (Feng et al., 2018; Kim et al., 2017a; Shuai et al., 2020; Sun et al., 2015; Wang et al., 2016b; Zheng et al., 2016b).

**Figure 1:**
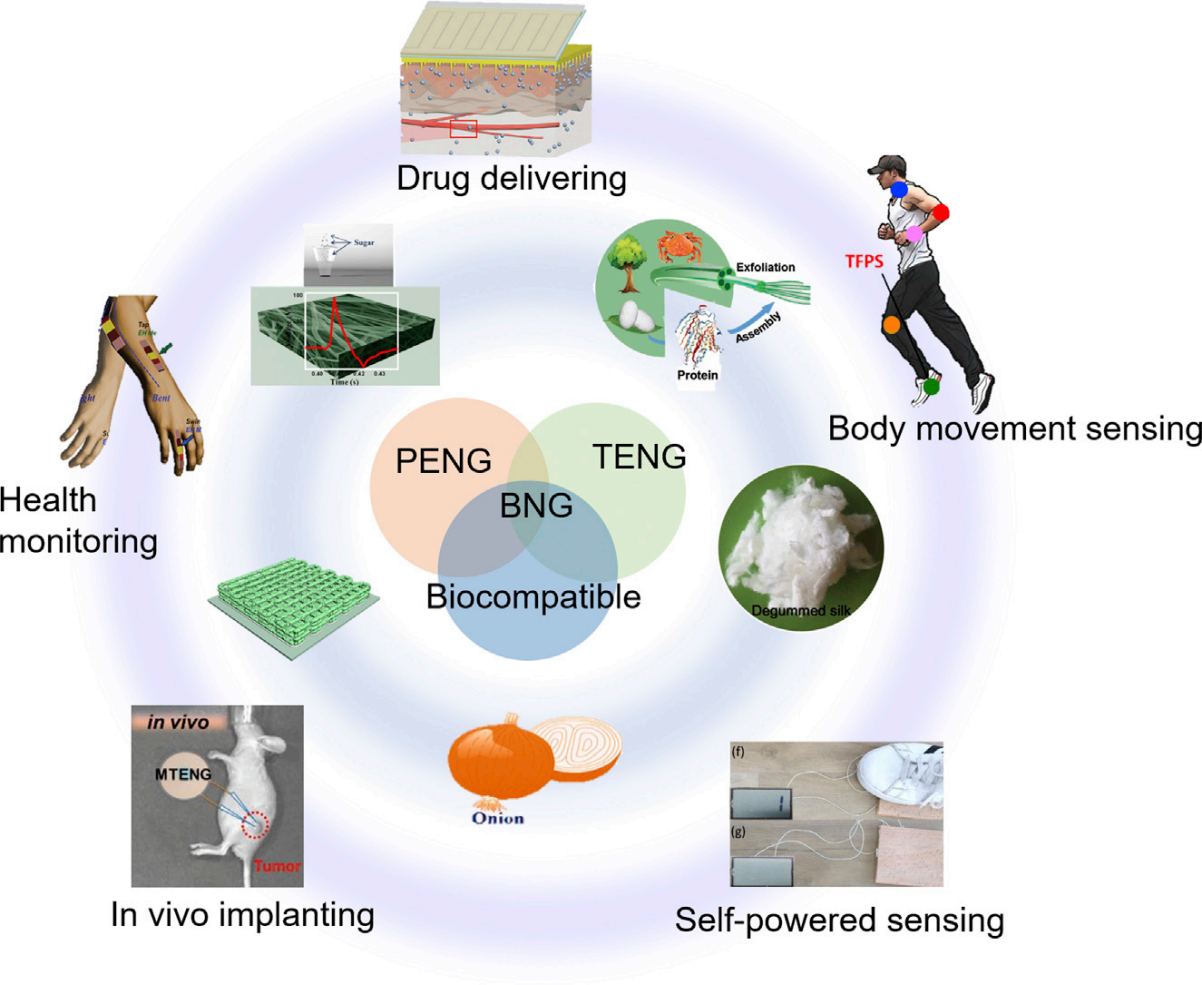
Biocompatible nanogenerators based on natural and synthetic materials and their applications in electronic skins, personal electronics, and other *in vitro* and *in vivo* biomedical applications

This review article focuses on the recent development of biocompatible nanogenerators that include PENGs, TENGs, and other nanogenerators. Figure 1 provides an overview of this article. In the first part, we reviewed the fundamentals of nanogenerators. In the second part, we introduced natural and synthetic biocompatible materials as energy conversion materials and different techniques for device fabrication. In the third part, we discussed various applications enabled by biocompatible nanogenerators and the application-specific requirements. At last, we highlighted the challenges faced by current biocompatible nanogenerators and the outlook of future research directions in this field.

## Development of nanogenerators

The large-scale mechanical energy in wind and river has long been an important source for human to acquire electricity. Scientists have tapped into a new energy source from the microscale and ubiquitous mechanical energy in the environment since the discovery of PENGs and TENGs. The awareness of environment protection and the needs for biomedical applications promoted the development of biocompatible nanogenerators that have the potential in improving the quality of our life.

### Piezoelectric nanogenerators

**Figure 2:**
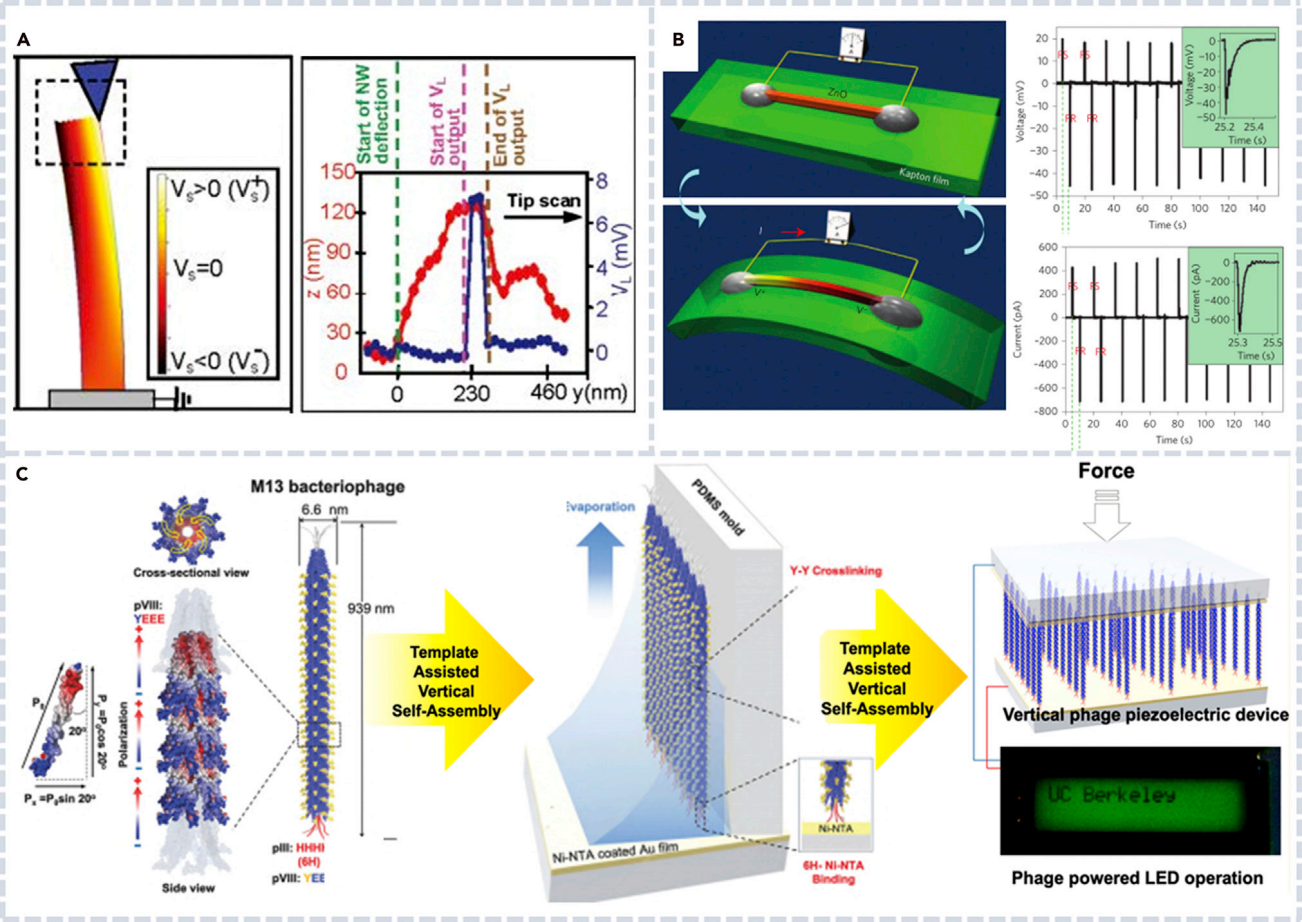
Working mechanism and related devices of PENGs (A) Potential distribution of a ZnO nanowire as it was bent by an atomic force microscope (AFM) tip (left) and line profiles from the topography (red) and output voltage (blue) images as the AFM tip scanned across the top of the nanowire (right) (Wang and Song, 2006). (B) The schematic and outputs of an alternating current PENG based on a single ZnO nanowire that was fixed on a flexible substrate (Yang et al., 2008). (C) Schematic of piezoelectricity of M13 bacteriophage and a PENG based on vertical phase arrays (Lee et al., 2019).

A PENG can directly convert ambient mechanical energy into electricity through the piezoelectric effect. The piezoelectric effect refers to the creation of polarization charges in a material when it is stressed, and the PENG uses the polarization potential to drive the current flow through the external circuit to realize the mechanical-electrical energy conversion. Wang et al. successfully demonstrated the energy conversion in ZnO nanowires (NWs) in 2006 (Figure 2A), which set the foundations for the development of PENG devices (Wang and Song, 2006). When ZnO NWs were bent, a strain field and charge separation were produced because of the coupling of piezoelectric and semiconducting properties in ZnO. The rectifying characteristic of the Schottky barrier between the atomic force microscope metal tip and NWs resulted in the direct current (DC) power generation. In 2007, Wang et al. developed an NW array-based device which produced continuous DC output as the NWs were excited by ultrasonic waves (Wang et al., 2007). However, the output was limited by the potential difference across the diameter of a bent NW. Yang et al. overcame the limit with a new design of a PENG that was based on a single NW fixed to a flexible substrate. The device generated an alternating current (AC) power output (Figure 2B), and it was sometimes called an AC nanogenerator (Yang et al., 2008). Thanks to its much higher voltage than that of a DC nanogenerator (Wang et al., 2007), thereafter, the AC nanogenerator dominated the development of nanogenerators. Zhu et al. produced a PENG using ZnO NW arrays and the generated AC power successfully lighted up a commercial light-emitting diode (LED) (Zhu et al., 2010).

The great success of the ZnO-based PENG inspired the investigation of nanogenerators with piezoelectric biomaterials and advanced significantly the development of biocompatible nanogenerators. A PENG based on M13 bacteriophage was invented (Lee et al., 2019). As described in Figure 2C, the device was based on the template-assisted vertical self-assembly of the bacteriophage, and the output power was used to operate a liquid crystal display with LED backlight. *In vitro* and in vivo PENGs have been discovered to harvest biomechanical energy (Li et al., 2010; Zhang et al., 2015). Yu et al. implanted a PENG under the skin of a rodent, and no toxicity or incompatibility sign was found during 6 weeks of operation (Yu et al., 2016).

### Triboelectric nanogenerators

**Figure 3:**
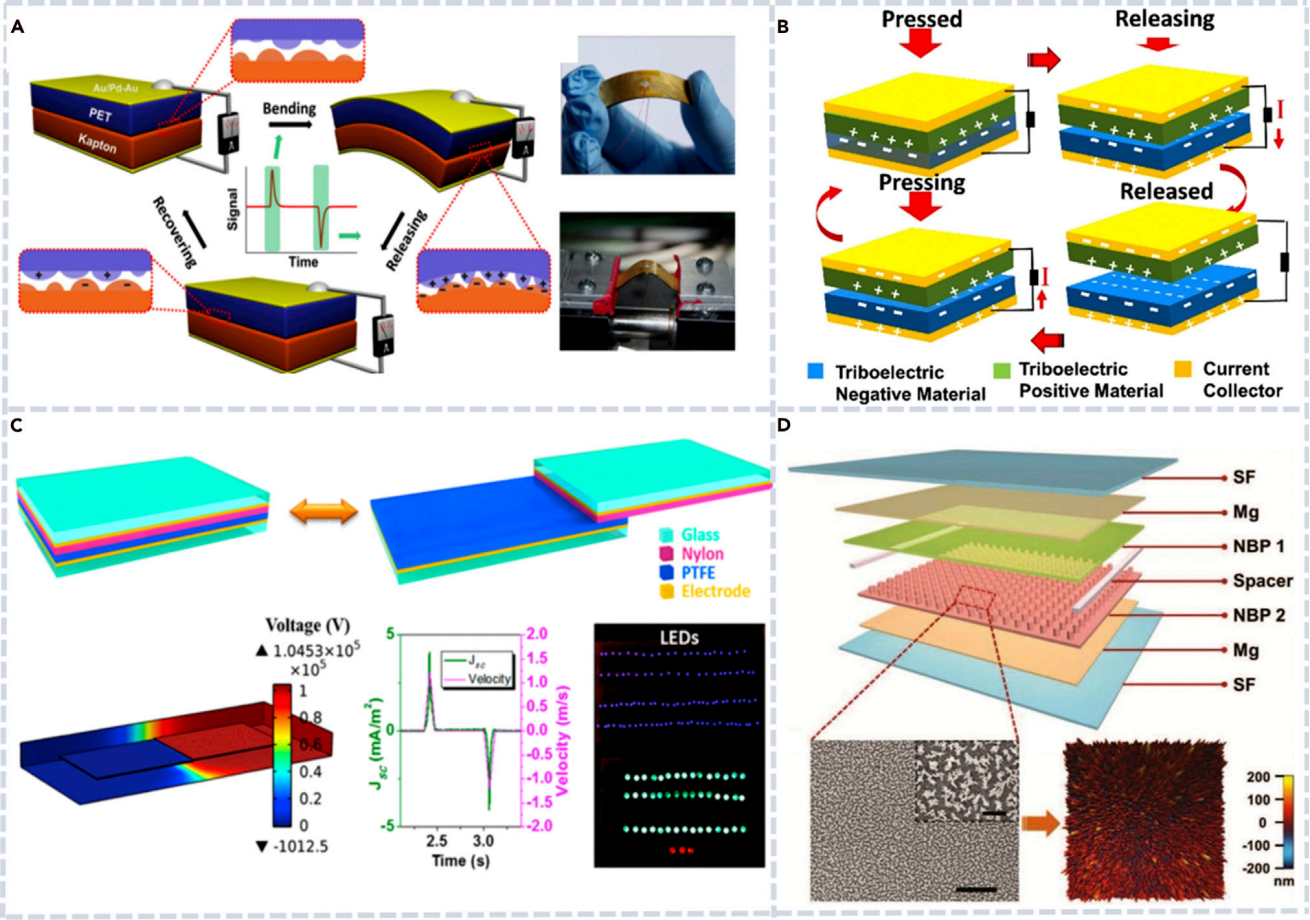
Working mechanism and related devices of TENGs (A) The first TENG that was composed of a Kapton film and a polyester film (Fan et al., 2012). (B) The flow chart of the contact-separation working mode of a TENG (Parida et al., 2019). (C) The schematic diagram of in-plane sliding mode of a TENG. Both the computation and the measurements showed good performance of the TENG that eventually drove hundreds of LED bulbs (Wang et al., 2013). (D) Structure diagram of a TENG based on bioabsorbable natural materials (Jiang et al., 2018).

Fan et al. reported the first energy harvester based on the triboelectric effect and electrostatic induction, and the device was later called a TENG (Fan et al., 2012). The device was composed of a Kapton film and a polyester film that were stacked together and had metal electrodes deposited on their back sides (Figure 3A). The so-called vertical contact-separation working mode is shown in Figure 3B. Two thin polymer films contact and separate when a mechanical force is applied and released. Meanwhile, the charges generated on the contacting surfaces of two different materials drive the electrons in the external circuit to flow back and forth; thus, the TENG converted mechanical energy into electricity. The TENG generated a maximum output voltage and current signal up to 3.3 V and 0.6 mA, respectively, and the power was high enough to directly drive an LED.

In addition to the contact-separation mode, the in-plane sliding mode was later found by Wang et al. (Figure 3C) (Wang et al., 2013). When the top and the bottom triboelectric materials contacted completely, positive and negative charges were generated on the contacting surfaces. When contacting surfaces were moved in the horizontal direction, an alternative electron flow was produced. The device generated an open-circuit voltage of 1300 V, a short-circuit current density of 4.1 mA/m^2^, and a peak power density (PD) of 5.3 W/m^2^. The energy produced by the TENG was used to drive hundreds of LED bulbs. This working mode was later used to fabricate devices with sliding cylinders and rotating discs (Bai et al., 2013; Jing et al., 2014; Lin et al., 2013). Bai et al. designed a cylindrical TENG to harvest mechanical energy from the rotational motion (Bai et al., 2013). The in-plane sliding mode shows superior device performance and greatly expands the application range of TENGs.

Later, the single-electrode mode was proposed to overcome the limitation of above two modes that required two electrodes to form a directional flow of electrons in the circuit (Lin et al., 2014). It consists of a ground electrode and a free-moving triboelectric layer. The potential difference is produced by contacting and separating periodically triboelectric layer, which results in the flow of electrons between the electrode and ground (Khandelwal et al., 2020). The freestanding mode was then developed from the single-electrode mode, which is composed of a charged layer and two symmetric electrodes (Zhu et al., 2014). Reciprocating motion of the charged layer between two electrodes without contacting leads to the change of the potential. In order to balance the potential difference, electrons flowed back and forth between the two electrodes through the external circuit load. The single-electrode mode and the freestanding mode showed special advantages in some application conditions, such as human-machine interface and flowing liquid.

The discovery of four working modes enabled TENGs to enter a rapid development stage. Various types of TENGs with new designs and different frictional materials were developed. During the process of designing the TENGs, material surface properties, device structure, and environmental effects had been emphasized. Besides motion parameters, temperature and humidity also affect the output of TENGs. In order to establish a unified standard to evaluate the output performance of different kinds of TENGs, Zi et al. proposed a standardized method to calculate the figure of merit (FOM). The FOM reflected the actual output capacity of TENGs. Meanwhile, the standardized method and FOM were also successfully applied to the poly(vinylidene fluoride) (PVDF) film-based PENGs (Xia et al., 2019; Zi et al., 2015).

As the application of nanogenerators extends to health monitoring and other human tissue related areas, the environment requires materials used in nanogenerators must be biocompatible. Implantable medical devices powered by a TENG are considered as a transformative technology for human health. Jiang et al. fabricated a bioabsorbable natural-material-based TENG via natural polymers (Figure 3D) (Jiang et al., 2018). The device was suitable for in vivo biomedical studies due to their biodegradable and bioresorbable property. Most importantly, it provides an effective method for the treatment of some heart diseases such as bradycardia and arrhythmia.

### Other nanogenerators

**Figure 4:**
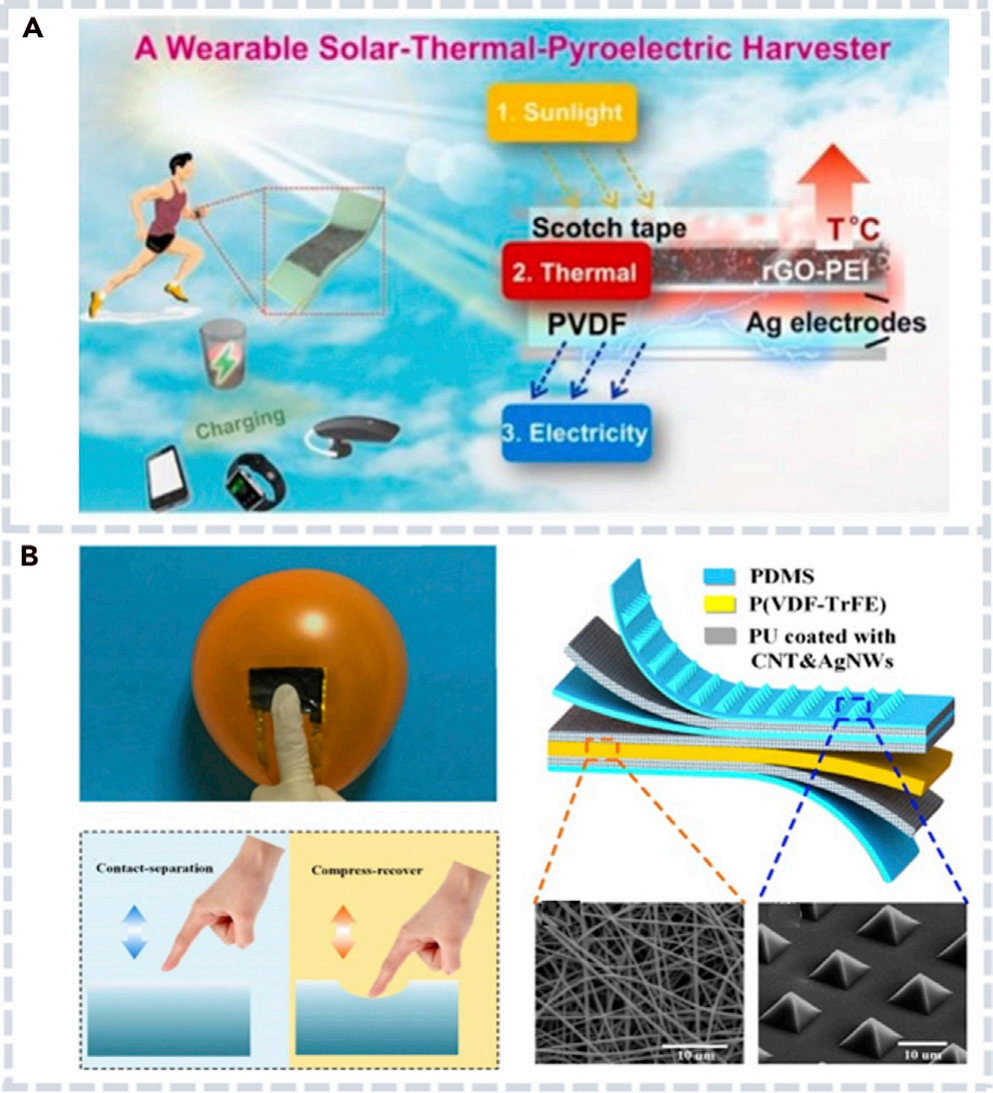
A pyroelectric nanogenerator and a hybrid nanogenerator (A) Application of a sunlight-triggered pyroelectric nanogenerator as a power source for wearable electronics (left) and the working mechanism of the nanogenerator (right) (Li et al., 2020a). (B) A flexible hybrid nanogenerator that can harvest energy from different motions (left) and the structure of the device (right) (Chen et al., 2017b).

In addition to PENGs and TENGs, devices (pyroelectric nanogenerators, electromagnetic generators, solar cells, and electrochemical cells) with other working mechanisms have also been invented. They can convert thermal, magnetic, solar, and chemical energy into electricity. For example, a pyroelectric nanogenerator was developed to recover the waste heat through the pyroelectric effect (Yang et al., 2012a, 2012b). The device took advantage of the anisotropic polarization in ZnO NWs to drive the electrons to flow, which was caused by the temperature fluctuation with the time. Li. et al. reported a highly efficient sunlight-triggered pyroelectric nanogenerator that was integrated in an outdoor bracelet (Figure 4A) (Li et al., 2020a).

Integrating different nanogenerators into one hybrid device is another way to harvest different forms of environmental energy (Chen et al., 2017a; Jurado et al., 2020; Wang et al., 2020a, 2020b). Among them, hybrid triboelectric-piezoelectric hybrid nanogenerators have been successfully prepared and widely applied in various fields. Chen et al. combined a poly(vinylidene fluoride-co-trifluoroethylene) (P(VDF-TrFE))-based TENG and a polydimethylsiloxane (PDMS)-based PENG to form a multilayer hybrid nanogenerator (Figure 4B) (Chen et al., 2017b). The device can be attached to the belly or wrist of a human body for monitoring physiological signals, which has a broad application potential in self-powered health monitoring systems. Vu Nguyen et al. simplified the structure of a hybrid nanogenerator by integrating a peptide-based PENG with a single-electrode TENG. By utilizing the friction charge generated by the TENG, the output performance of the PENG can be improved (Nguyen et al., 2017). Other hybrid energy harvesters have also been proposed, such as hybridized electromagnetic-triboelectric nanogenerators, electromagnetic-piezoelectric-triboelectric hybrid nanogenerator, and the hybridization of solar cells with a TENG or electromagnetic nanogenerator (Guo et al., 2018; Li et al., 2019a; Singh and Khare, 2018).

## Materials and fabrication methods for biocompatible nanogenerator technologies

**Figure 5:**
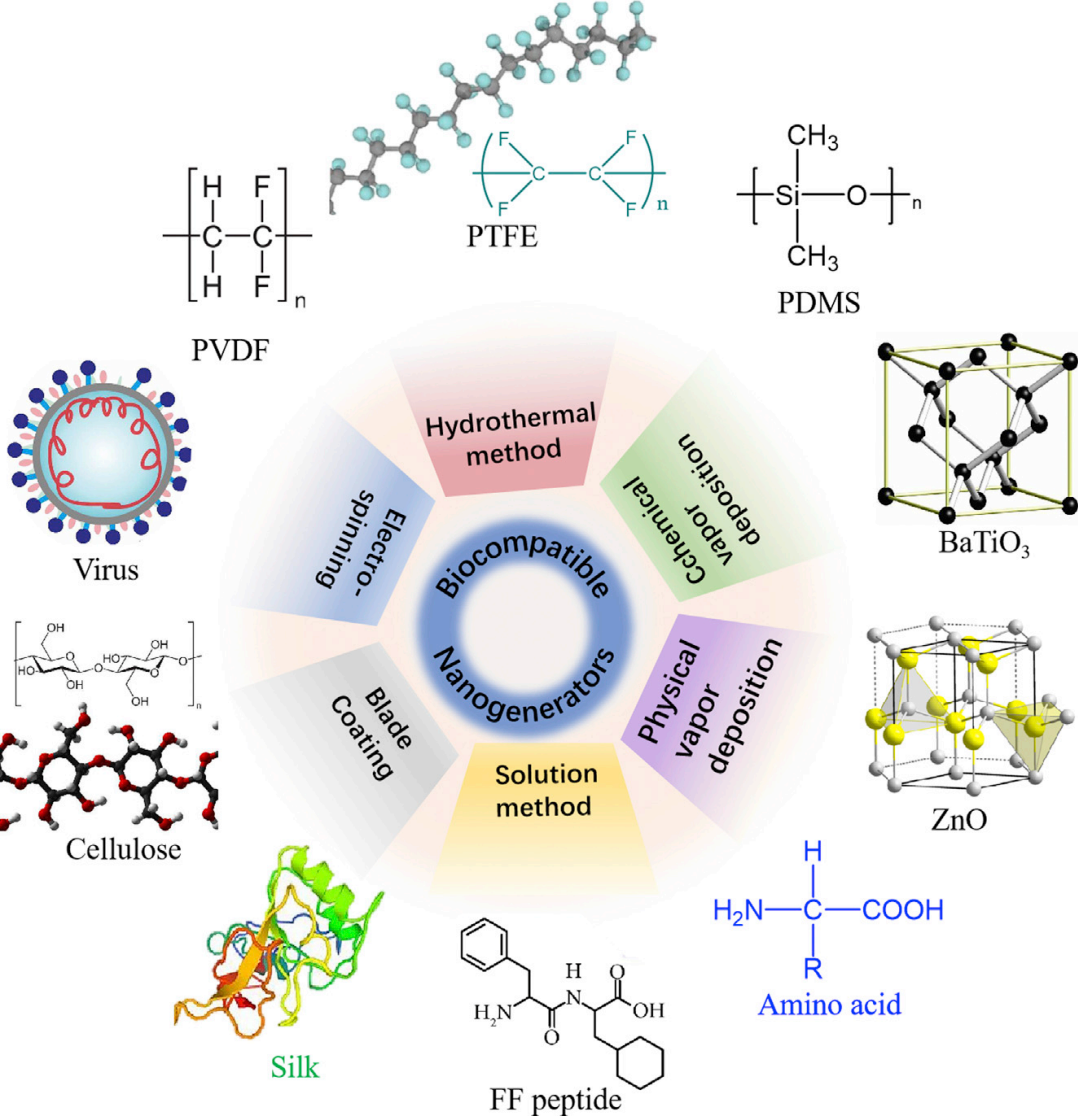
Representative materials and fabrication methods for biocompatible nanogenerators

To improve the performance of biocompatible nanogenerators, both materials and fabrication methods have been widely explored in recent years. The functional material used in biocompatible nanogenerators includes polymers, biomolecules, and inorganic materials (Figure 5). Among which, polymers show excellent durability and reliability, while biomolecule-based materials show the best biocompatible potential. For constructing biocompatible nanogenerators, electrospinning, aqueous dispersion, direct writing technology, hydrothermal synthesis, and other techniques are widely studied.

### Polymer-based nanogenerators

#### PVDF-based materials

**Figure 6:**
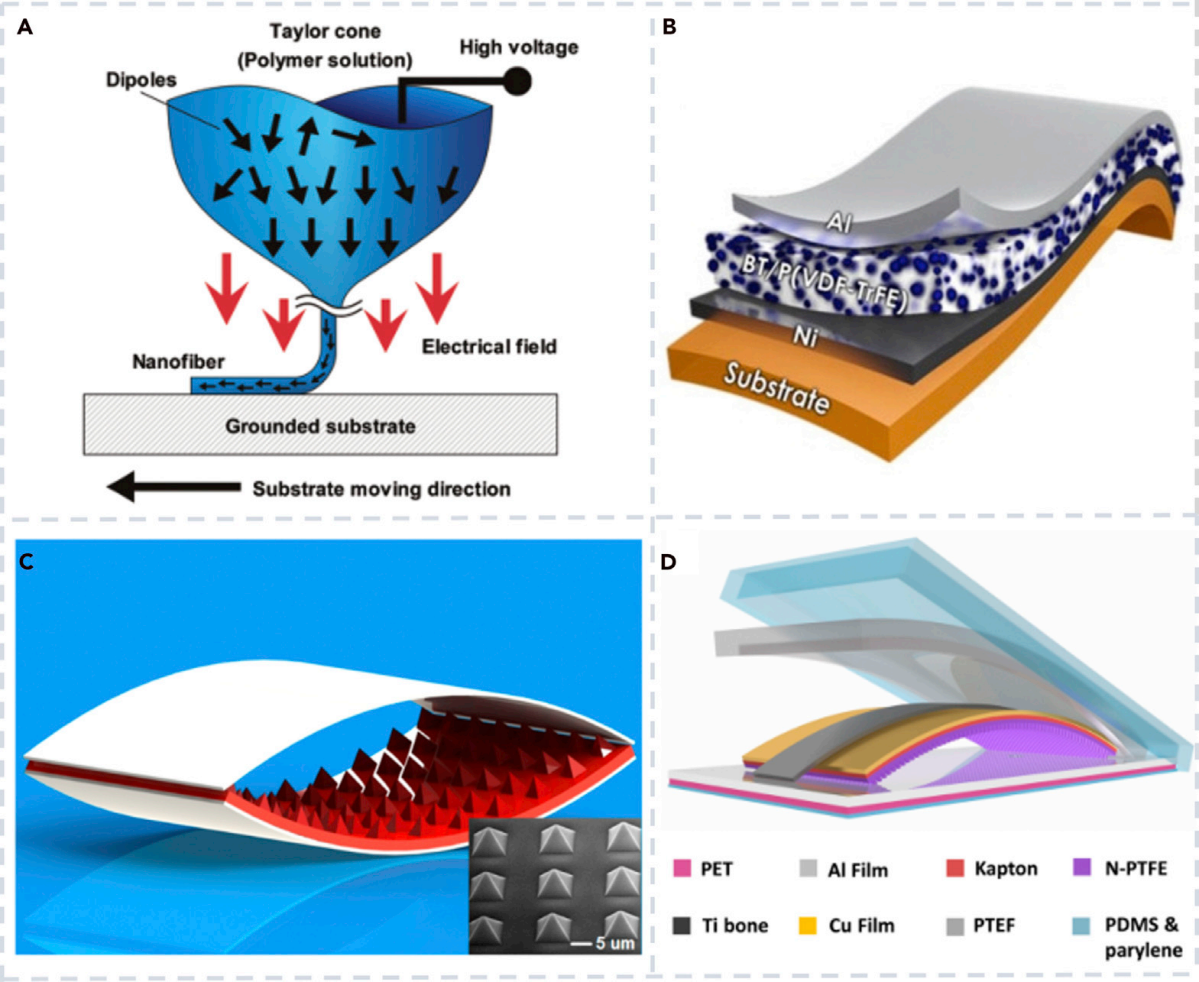
Biocompatible nanogenerator based on different polymers (A) Fabrication of piezoelectric PVDF nanofibers for a PENG by combing near-field electrospinning, direct-write, mechanical stretching, and *in situ* electrical poling (Chang et al., 2010). (B) Schematic of a PENG based on BT/P(VDF-TrFE) composites (Siddiqui et al., 2015). (C) Schematic illustration of a flexible arch-shaped TENG (Tang et al., 2015). (D) Schematic diagram of a biocompatible TENG based on nanostructured PTFE (Zheng et al., 2016a).

PVDF and P(VDF-TrFE) are the most studied polymers and good candidates for the fabrication of biocompatible nanogenerators due to their good piezoelectricity, flexibility, biocompatibility, and processability. Chang et al. created PVDF nanofiber-based nanogenerators by using a direct-write technology with a near-field electrospinning process (Chang et al., 2010). Figure 6A shows the schematic process of the nanogenerator fabrication. Siddiqui et al. improved the performance of a piezoelectric nanogenerator by imbedding barium titanate nanoparticles into P(VDF-TrFE) films (Siddiqui et al., 2015). The schematic of the nanocomposite PENG is shown in Figure 6B. Thanks to the enhanced piezoelectricity of P(VDF-TrFE) by barium titanate nanoparticles, the nanocomposite PENG produced an output voltage and output PD as high as that of lead-containing PZT-based PENGs.

#### PDMS-based materials

While most polymers are not piezoelectric materials and not good for a PENG, many polymers have been successfully used for a TENG. PDMS has been used in various biocompatible nanogenerators due to its transparency, hydrophobicity, and excellent electronegativity (Kim et al., 2018a; Patnam et al., 2020). He et al. proposed a PDMS/MXene composite-based flexible single-electrode biocompatible TENG. Wang et al. fabricated an arch-shaped highly flexible device, which used PDMS and indium tin oxide as the friction materials (Figure 6C) (Tang et al., 2015). The pyramid array-patterned PDMS film was designed to enhance the TENG's current output of the plain film.

#### PTFE-based materials

Compared with other materials such as polyurethane, nylon (polyamide), and polyethylene terephthalate, PTFE has the advantages of high durability and water repellency (Choi et al., 2019; Singh and Khare, 2018; Wang et al., 2020a). Guo et al. developed a PTFE-based TENG for an automobile safety system, which showed high durability of one million working cycles (Liu et al., 2020). Zheng et al. used PTFE and aluminum (Al) to design and fabricate a biocompatible TENG (Zheng et al., 2016a). PDMS was used as the packaging material in the device for enhanced biocompatibility and liquid leakage prevention (Figure 6D). The biocompatible nanogenerator was placed in a body fluid environment for analog detection, and its output voltage and current were 60 V and 12 μA, respectively. After integrating the nanogenerator with the data collection, data processing, and wireless transmission circuit, the system worked with the power harvested from the heart beating of an animal and realized wireless monitoring of the biological indicators of the heart.

### Biomolecule-based nanogenerators

#### Cellulose-based materials

**Figure 7:**
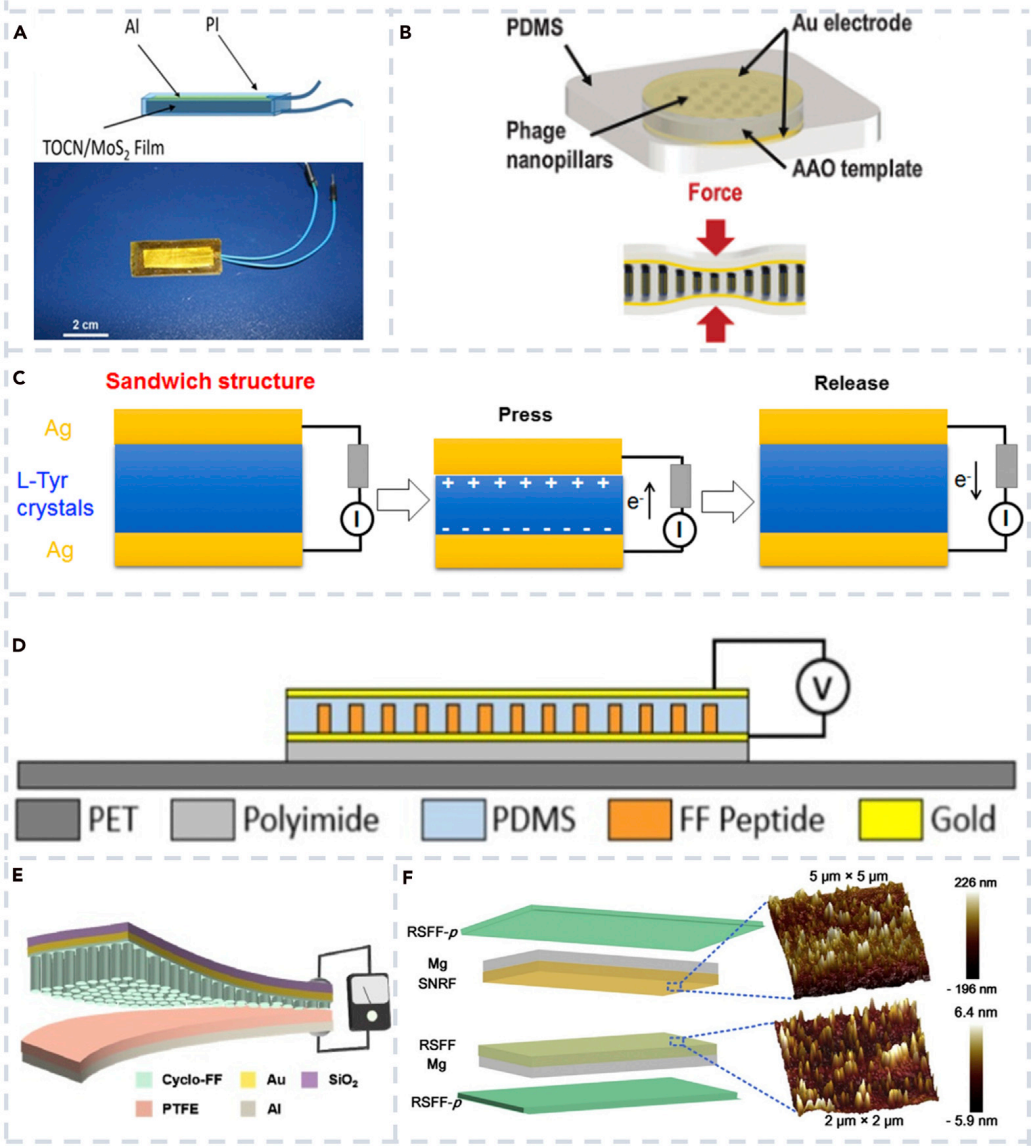
Biocompatible nanogenerator based on different biomolecules (A) Schematic diagram of a TOCN/MoS_2_ nanogenerator (Wu et al., 2021). (B) Schematic of a phage nanopillar-based nanogenerator (Shin et al., 2015). (C) The sandwich structure of an L-Tyr crystal-based nanogenerator and its energy conversion process (Ji et al., 2019). (D) Schematic of the FF peptide nanogenerator attached to a PET beam (Jenkins et al., 2018). (E) Schematic view of a TENG based on Cyclo-FF nanowire arrays (Park et al., 2019). (F) Schematic illustration of a biocompatible TENG using silk nanofibrils (Niu et al., 2020).

Cellulose-based materials are used for nanogenerators due to their flexibility, low cost, and simple manufacture techniques (Guo et al., 2017; Nie et al., 2021; Shen et al., 2020; Wu et al., 2018; Zhang et al., 2020a, 2020b). Wu et al. reported that bionanocomposite films consist of 2,2,6,6-tetramethylpiperidine-1-oxyl-oxidized cellulose nanofibril (TOCN) and molybdenum disulfide (MoS_2_) nanosheets by aqueous dispersion (Wu et al., 2021). The structural diagram of the TOCN/MoS_2_ nanogenerator is shown in Figure 7A. The result showed that the composite material had good mechanical properties. The highest Young's modulus was 8.2 GPa, and the tensile strength was 307 MPa. The nanogenerator made from the TOCN/MoS_2_ composite film was showed to collect mechanical energy in the environment. Recently, Zhang et al. developed a cellulose-based fully green TENG with a PD of above 300 W m^−2^, which is a new record for green-material-based TENGs (Zhang et al., 2020a). Due to the high stability of cellulose, cellulose-based nanogenerators often have high durability with working cycles from 30 thousands (Zhang et al., 2020a) to 50 thousands (Wang et al., 2017).

#### Virus-based materials

Virus is a special organism with a very simple structure consisting of only a long nucleic acid chain and a protein coat (Jeong et al., 2013; Lee et al., 2012, 2019). One of the most famous viruses is the M13 phage. Shin et al. developed bioinspired nanogenerators based on vertically aligned phase nanopillars (PNP) through enforced infiltration (Figure 7B) (Shin et al., 2015). The electrical output of the vertically aligned PNP-based PENG was up to ∼2.6-fold greater than that of the laterally assembled phage-based nanogenerator. The reason may be that the vertically aligned PNP has higher axial elasticity and better piezoelectric properties.

#### Acid amino-based materials

Amino acids are a class of organic compounds containing amine groups (-NH_2_), carboxyl groups (-COOH), and specific types of side chains (R groups). They have good water solubility. There are 20 kinds of amino acids in human body (Guerin et al., 2018; Kim et al., 2020). Ji et al. investigated the self-assembly of three amino acids L-phenylalanine (L-Phe), L-tyrosine (L-Tyr), and L-DOPA, which have very similar chemical structures (Ji et al., 2019). They created a biocompatible PENG based on L-Tyr crystal films. The sandwich structure of the L-Tyr crystal-based biocompatible nanogenerator is shown in Figure 7C. By applying a pressure of 31 N, the nanogenerator produced a high and stable power output. The open-circuit voltage reached 0.5 V and short-circuit current reached 35 nA. These pioneering works inspired future exploration of the use of amino acids in biocompatible nanogenerators.

#### Peptide-based materials

Diphenylalanine (FF) peptide shows great promise in energy harvesting due to its good mechanical properties, piezoelectricity, and biocompatibility (Lee et al., 2018; Tao et al., 2019a, 2019b, 2020). Jenkins et al. explored the piezoelectricity of FF peptides using multi-physics finite element models (Figure 7D) (Jenkins et al., 2018). Finite element analysis showed that the FF peptide NW-based PENG can produce higher voltage than ZnO and BTO NWs under the same force. The output voltage of a peptide-based flexible biocompatible PENG was in good agreement with the results of finite element analysis. Park et al. created a biocompatible TENG based on vertically aligned cyclo-diphenylalanine (Cyclo-FF) NWs (Figure 7E) (Park et al., 2019). The lyophilized Cyclo-FF powder was evaporated and self-assembled on a substrate to form vertically aligned NWs. The dimension of the Cyclo-FF NWs was controlled through thermal evaporation process. The study showed that the peptide nanostructures were stable under different moisture. The prepared generator generated an open-circuit voltage and short-circuit current of ∼350 V and ∼10 μA, respectively. The maximum output power of the nanogenerator is 73.7 mW/m^2^. Yang's group has explored widely on peptide-based biocompatible PENGs (Jenkins et al., 2018; Nguyen et al., 2016; 2017; Zhang et al., 2014). Different growth methods of several peptides were developed to construct biocompatible PENGs, and finite element modeling was used to guide the design of the peptide-based nanogenerator. The peptide-based PENG also showed high durability with 2,500–5,000 testing cycles (Nguyen et al., 2016; Tao et al., 2019a).

#### Silk-based materials

Silk nanofibrils (SNFs) have excellent biocompatibility, flexibility, and strength (Sheng-You et al., 2020; Niu et al., 2020; Wen et al., 2019). Niu et al. reported a biocompatible TENG based on silk nanoribbons using a nascent SNR film and regenerative silk fibroin film (Figure 7F) (Niu et al., 2020). The nanogenerator produced a maximum voltage, current, and PD of up to 41.64 V, 0.5 μA, and 86.7 mW/m^2^, respectively. The materials used in the biocompatible TENG include silk and Mg that are fully biodegradable and biocompatible. In addition, the TENG showed high durability with over 3,000 cycles of tests. Karan et al. used a natural spider-silk-constructed biocompatible PENG, which showed high output and high durability with up to 30,000 tests cycles (Karan et al., 2018). Silk-based biocompatible TENG is expected to be a popular energy source with potential applications in implantable self-powered electronic devices, pacemakers, and implanted sensors.

### Inorganics based nanogenerator

#### ZnO-based materials

**Figure 8:**
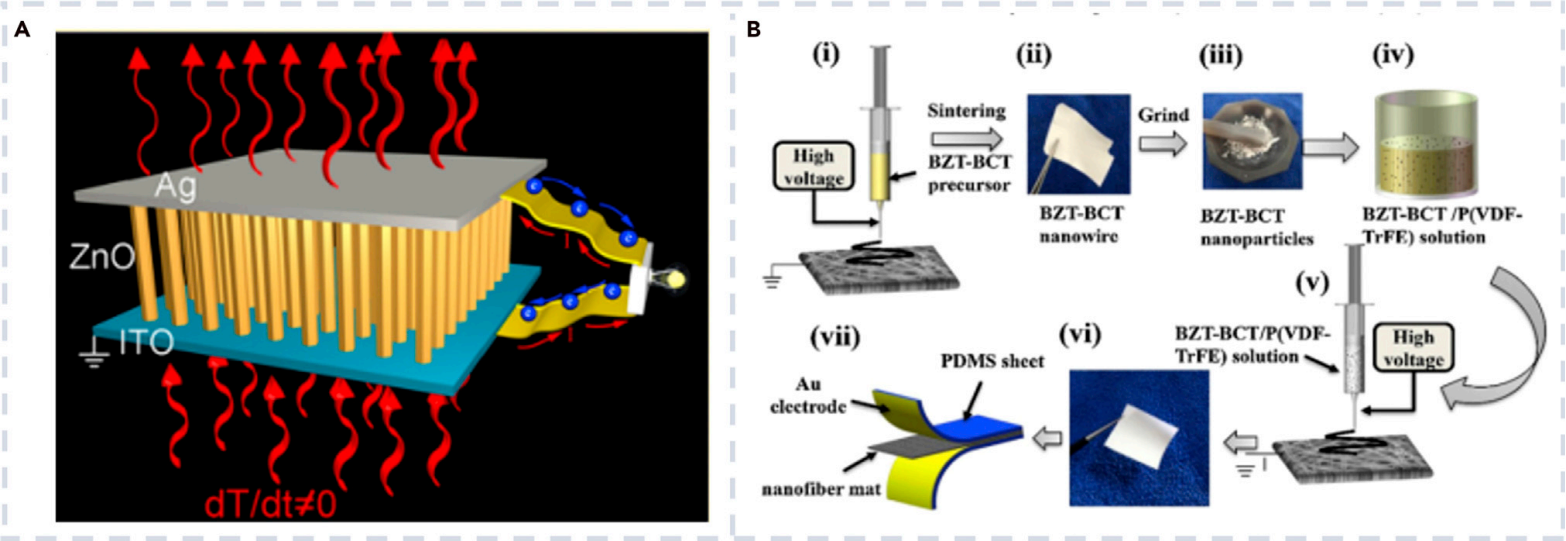
Biocompatible nanogenerator based on different inorganics (A) The structure of a pyroelectric nanogenerator (Yang et al., 2012a). (B) Fabrication process of a BZT-BCT/P(VDF-TrFE)-based nanogenerator (Liu et al., 2020).

ZnO has abundant nanostructures, such as NWs (Wang and Song, 2006), nanorings (Kong et al., 2004), and nanohelices (Hao et al., 2017). ZnO nanostructures exhibit excellent piezoelectricity and biocompatibility (Li and Wang, 2017; Zhang et al., 2014; Zhou et al., 2006; Zhu et al., 2010). Wang and co-workers leaded the research of ZnO-based PENGs (Wang and Song, 2006). The PENG converted random mechanical energy into electric energy. Yang et al. fabricated a pyroelectric nanogenerator and demonstrated the first application of ZnO NW arrays in converting thermal energy into electrical energy using Seebeck effect (Figure 8A) (Yang et al., 2012a). The biocompatible pyroelectric nanogenerator showed good stability, and the characteristic coefficient of heat flow conversion into electricity was estimated to be ∼0.05–0.08 V m^2^/W.

#### Other lead-free materials

The materials used in bioelectronics usually need to contact with human body, so their biosafety and biocompatibility have received great attention. Lead-free materials such as BTO and potassium sodium niobate have been explored to construct nanogenerators (Bairagi and Ali, 2020; Li et al., 2020b). Liu et al. developed lead-free PENG based on 0.5Ba(Zr_0.2_Ti_0.8_)O_3_-0.5(Ba_0.7_Ca_0.3_)TiO_3_ (BZT-BCT) and P(VDF-TrFE) nanofibers via an electrospinning method (Liu et al., 2020). Figure 8B shows the fabrication process of BZT-BCT/P(VDF-TrFE)-based nanogenerators. The output voltage was enhanced significantly with 40% BZT-BCT content. The nanogenerator generated electricity with tiny human motions and showed high durability with 5,000 testing cycles.

## Applications of biocompatible nanogenerators

Biocompatible nanogenerators are good candidates to supply power to biomedical or skin-contact electronic devices. Because of their low cost and environmental friendliness, biocompatible nanogenerators have been extensively used in many areas (Table 1). In this section, we discuss the representative works regarding energy harvesting, biosensing, health monitoring, drug delivering, and *in vivo* implanting biomedical applications assisted with biocompatible nanogenerators.

**Table 1:** Summary of representative biocompatible nanogenerators

	Materials	Advantages	Performance	Application	Ref.
PENG	ZnO	Simple, effective	2.03 V, 107 nA, 11 mW/cm^3^	Self-powered systems	(Zhu et al., 2010)
	Spider silk	Biocompatible, ultra-sensitive	21.3 V, 0.68 μA, 4.56 μW/cm^2^	Physiological signal monitoring	(Karan et al., 2018)
	Diphenylalanine peptide	Biocompatible	1.4 V, 3.3 nW/cm^2^	Biomechanical energy harvesting	(Nguyen et al., 2016)
	Boron nitride nanosheet, PDMS	Transparent, biocompatible	22 V, 75 nA, 106 μW/cm^2^	Body movement sensing	(Kim et al., 2018b)
	Wood sponge	Low-cost, biodegradable, biocompatible, highly compressible	0.69 V, 7.1 nA	Wearable human motion monitoring	(Sun et al., 2020a)
	Onion skin		18 V, 166 nA, 1.7 μW/cm^2^	Pacemakers, health care, speech recognition	(Maiti et al., 2017)
	AlN	Flexible, biocompatible	1.4 V, 1.6 μA,	Wearable energy harvesters	(Algieri et al., 2018)
	III-N thin-film	Biocompatible, durable	30 V, 6 μA, 167 μW	Energy harvesting	(Chen et al., 2019b)
TENG	Cellulose	Green, higher performance	736 V, 66.5 μA, 307 W/m^2^	Green TENGs for energy harvesting and self-powered sensing	(Zhang et al., 2020a)
	poly(lactic-co-glycolic acid (PLGA), PVA	Breathable, biodegradable, antibacterial	130 mW/m^2^, voltage response pressure sensitivity 0.011 kPa^−1^	Whole-body physiological signal and joint movement monitoring	(Peng et al., 2020)
	Chitosan	Biodegradable, flexible	13.5 V, 42 nA	Economical, biodegradation rate tunable	(Wang et al., 2018)
	PDMS, PDMS/multiwalled carbon nanotube (MWCNT)	Flexible, biocompatible	30 V, 130 μW	Lighting LED bulbs	(Zhu et al., 2016)
	TiO_2_ nanotube film	Wearable, stretchable, portable	44.6 mW/m^2^	Wearable motion sensor	(Zhang et al., 2018)
	PDMS, Al	Wearable, Biocompatible	33 μW	Drug delivery	(Wang et al., 2016b)
	Cellulose, PDMS	Biocompatible, biodegradable, sensitive	52 V	Humidity sensing	(Qian et al., 2019)
Hybrid nanogenerators	Silk fibroin, PVDF	Flexibility, multifunction,	500 V, 12 μA, 0.31 mW/cm^2^	Health monitoring	(Guo et al., 2018)
	Cellulose, BaTiO_3_/MWCNT	Simple structure, lightweight, green	18 V, 1.6 μA/cm^2^,	Dynamic pressure detection	(Li et al., 2019a)
	PDMS, PVDF, silver nanowires	Transparent, flexible, biocompatible	86 V	Healthcare monitoring	(Sun et al., 2018)

### Biocompatible nanogenerators as energy sources for sensors

**Figure 9:**
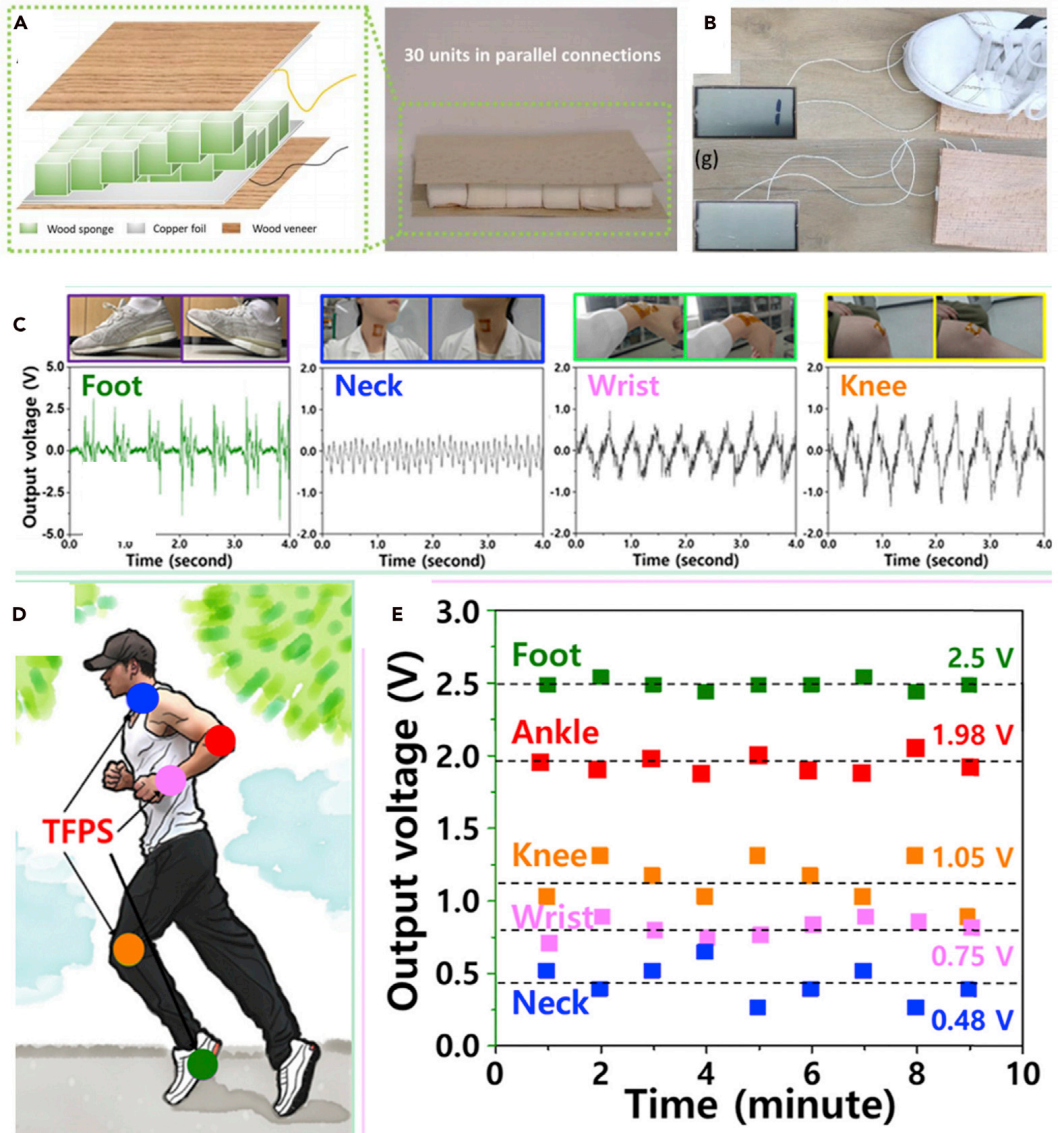
Biocompatible nanogenerators for energy harvesting (A and B) (A) A diagram of wood sponge-based PENG and (B) its application in a self-powered system (Sun et al., 2020a). (C–E) Flexible and transparent TENGs served as body movement sensors, which clearly distinguished motions of a foot, ankle, knee, wrist, and neck (Kim et al., 2018b).

Because biocompatible nanogenerators can readily generate electricity by scavenging energy from the environment, they have been widely used in self-powered systems (Alluri et al., 2017; Fan et al., 2016; Niu et al., 2020; Najjar et al., 2017; Saravanakumar et al., 2015; Sun et al., 2020a; Sun et al., 2020b; Wang et al., 2017). In addition to serving as a power source, biocompatible nanogenerators can also be used as a sensor, such as a pressure sensor or chemical sensor (Khandelwal et al., 2019; Kim et al., 2018b; Qian et al., 2019; Saravanakumar et al., 2015). Burgert et al. developed a low-cost, biocompatible, biodegradable, and highly efficient PENG based on wood sponge. The wood sponge PENG was able to generate a voltage of up to 0.69 V and a current of 7.1 nA. The PENG was used as a sustainable and renewable energy source for a wearable sensor to monitor human motions (Figures 9A and 9B) (Sun et al., 2020a). Kim et al. fabricated a self-powered ultraviolet photosensor by coupling a composite biocompatible nanogenerator to a photodetector (Saravanakumar et al., 2015). However, the sensor worked by combining the composite biocompatible nanogenerator with a ZnO NW photodetector (Saravanakumar et al., 2015). Kim et al. developed a biocompatible metal-organic framework (MOF)-based TENG and detected tetracycline through the high specific binding between the MOF ligand and tetracycline (Khandelwal et al., 2019). Sung et al. dispersed boron nitride nanosheets in poly-PDMS and fabricated a biocompatible TENG, which served as a flexible and transparent wearable body movement sensor (Figures 9C–9E) (Kim et al., 2018b).

Biocompatible TENGs based on natural materials, such as cellulose, have significant advantages over TENGs based on traditional materials to serve as certain sensors (Kim et al., 2017b; Sun et al., 2020b; Wang et al., 2017). By using an all-printing method, Qian et al. fabricated a biocompatible microhierarchical/nanohierarchical TENG based on cellulose, which acted as a self-powered mechanical sensor and humidity sensor simultaneously (Qian et al., 2019). The self-powered system detected the motion of fingers and legs and measured humidity accurately with sensing responsive ratio up to 5:1. By employing cellulose acetate and Kapton as the triboelectric layers, Prasad et al. developed a robust environmental friendly TENG which acted as a force sensor with a linear response over the force range of 0.24 N–5.2 N (Srither et al., 2018).

### Biocompatible nanogenerators for *in vitro* health monitoring and e-skin

**Figure 10:**
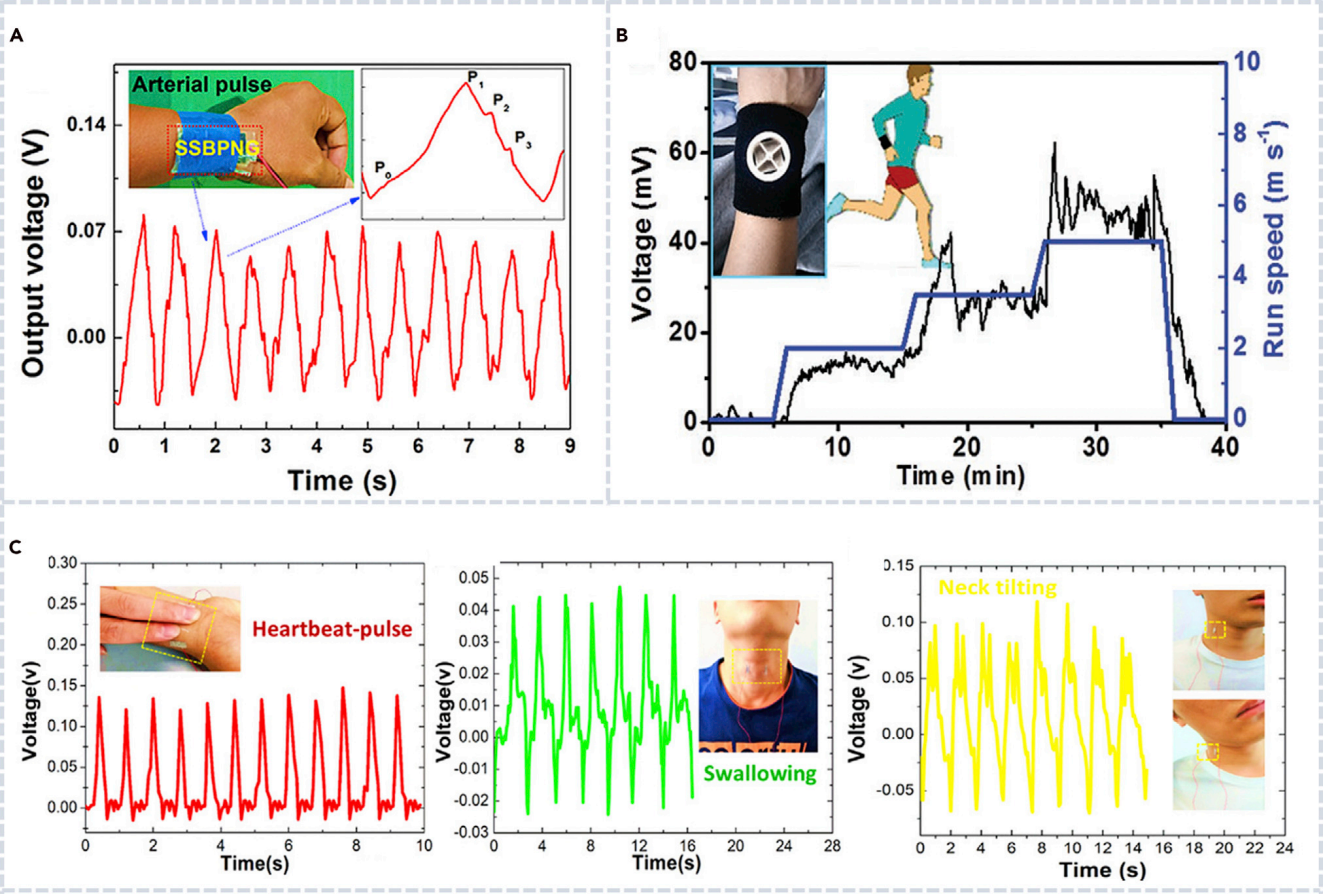
Biocompatible nanogenerators for *in vitro* health monitoring and an e-skin (A) Output voltage of a spider silk biocompatible PENG driven by arterial pulses (right) (Karan et al., 2018). (B) V_oc_ variation of a nanogenerator installing on spire lamella for perspiration-driven power supply and body motion sensor (Li et al., 2019b). (C) Voltage output of a transparent hybrid nanogenerator for monitoring heartbeat pulse, swallowing, and neck tilting (Sun et al., 2018).

*In vitro* applications of biocompatible nanogenerators require the precursor materials to be nontoxic and biocompatible. As mentioned earlier, cellulose (He et al., 2018; Kim et al., 2017b; Wang et al., 2017), silk protein (Jiang et al., 2019; Karan et al., 2018; Liu et al., 2017; Niu et al., 2020; Najjar et al., 2017), chitin (Li et al., 2019b), peptide (Yuan et al., 2019; Nguyen et al., 2016; 2017), and biocompatible polymers have been widely explored for constructing nanogenerators for health monitoring devices or e-skin applications (Rao et al., 2020; Peng et al., 2020). Khatua et al. developed a robust PENG based on biocompatible and biodegradable spider silk (Figure 10A). The device is ultra-sensitive toward arterial pulse (Karan et al., 2018). Li et al. developed a biological nanogenerator based on nanofibrils, which generated electricity from moist air flow in nature and showed a sensitive body kinematics sensing capability with additional biocompatibility, biodegradation, and antibacterial properties (Figure 10B) (Li et al., 2019b). Chen et al. developed a simple, transparent, flexible, and compatible triboelectric-piezoelectric-pyroelectric hybrid nanogenerator, which was attached to skin-like soft objects to monitor human physiological signals including heartbeat pulse, swallowing, and neck tilting. The hybrid nanogenerator showed great potential for cost-effective medical diagnostics and prognostication of cardiovascular, esophagus and Parkinson's disease (Figure 10C) (Sun et al., 2018).

The detection modes of the self-powered health monitoring devices or e-skin are of great importance to obtain high sensitivity and accuracy. Normally, there are three kinds of detection modes: amplitude mode, ratio mode, and frequency mode (Chen et al., 2019a). Amplitude mode can provide information for detecting various signals; however, it may be affected by environmental elements like temperature and humidity. Ratio mode needs two or more devices in the sensing system to compare; it can thus avoid distortion of external elements. Frequency mode depends on the waveform and frequency as a digital sensor, which can retain the time information. Different detection modes may be taken into account at the same time in order to obtain optimal results for specific functions.

### Biocompatible nanogenerators for *in vivo* implantable devices

**Figure 11:**
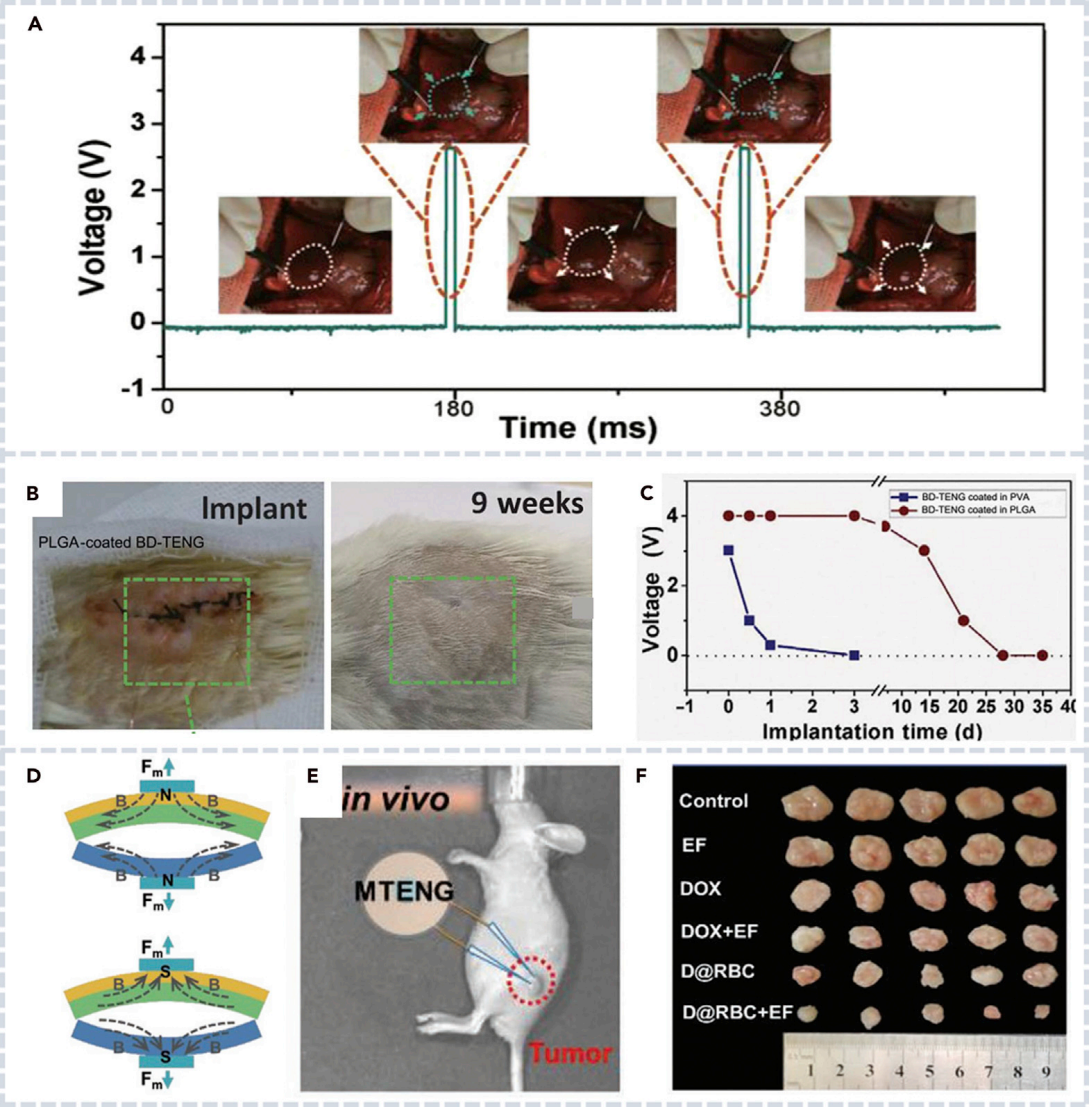
Biocompatible nanogenerators for *in vivo* implanting applications (A) Stimulation of the heart of a rat by a self-powered pacemaker (Zheng et al., 2014). (B) BD-TENG implanted in the subdermal dorsal region of an SD rat right after suture (left) and 9 weeks after suture (right) (Zheng et al., 2016b). (C) Electrical output of BD-TENGs at several time intervals after implantation (Zheng et al., 2016b). (D) Diagrams of magnetic TENGs. The two friction layers separate from each other due to magnetic repulsion (Zhao et al., 2019). (E) The sketch map of MTENG-controlled RBC DDS in the tumor-bearing nude mice (Zhao et al., 2019). (F) Image of the harvested tumors in various groups after 1 month (Zhao et al., 2019).

Biocompatibility is critical for *in vivo* applications of nanogenerators, and biodegradability is an important property for materials used in some implant applications. Wang et al. developed a PDMS-based TENG to generate electricity from rat's normal breathing. The TENG produced a PD of 8.44 mW m^−2^ and drove a pacemaker prototype to regulate heart rate of the rat (Figures 11A) (Zheng et al., 2014). Furthermore, Wang et al. developed a biodegradable TENG by using low-cost and commercially available materials to convert *in vivo* biomechanical energy into electric power for implantable medical devices (Zheng et al., 2016b). Nerve cell growth was successfully orientated by the TENG. *In vivo* 9-week implantation was achieved without significant inflammatory reaction, and the wound healed well (Figures 11B and 11C). Early reported TENGs usually faced the challenge of significantly decreased output when the devices were implanted due to the difficulties of detachment of two friction layers. Recently, Li et al. fabricated a magnet triboelectric nanogenerator which ensured the contact and detach cycle by magnetic repulsion between the two friction layers. Thus, the device maintained a high and consistent electricity output for a long time (Figures 11D). After implanted into an sprague dawley (SD) rat, the device enabled a self-powered electric field-controlled drug delivery system (DDS) for cancer therapy (Figures 11E and 11F) (Zhao et al., 2019).

### Biocompatible nanogenerators for drug delivery

**Figure 12:**
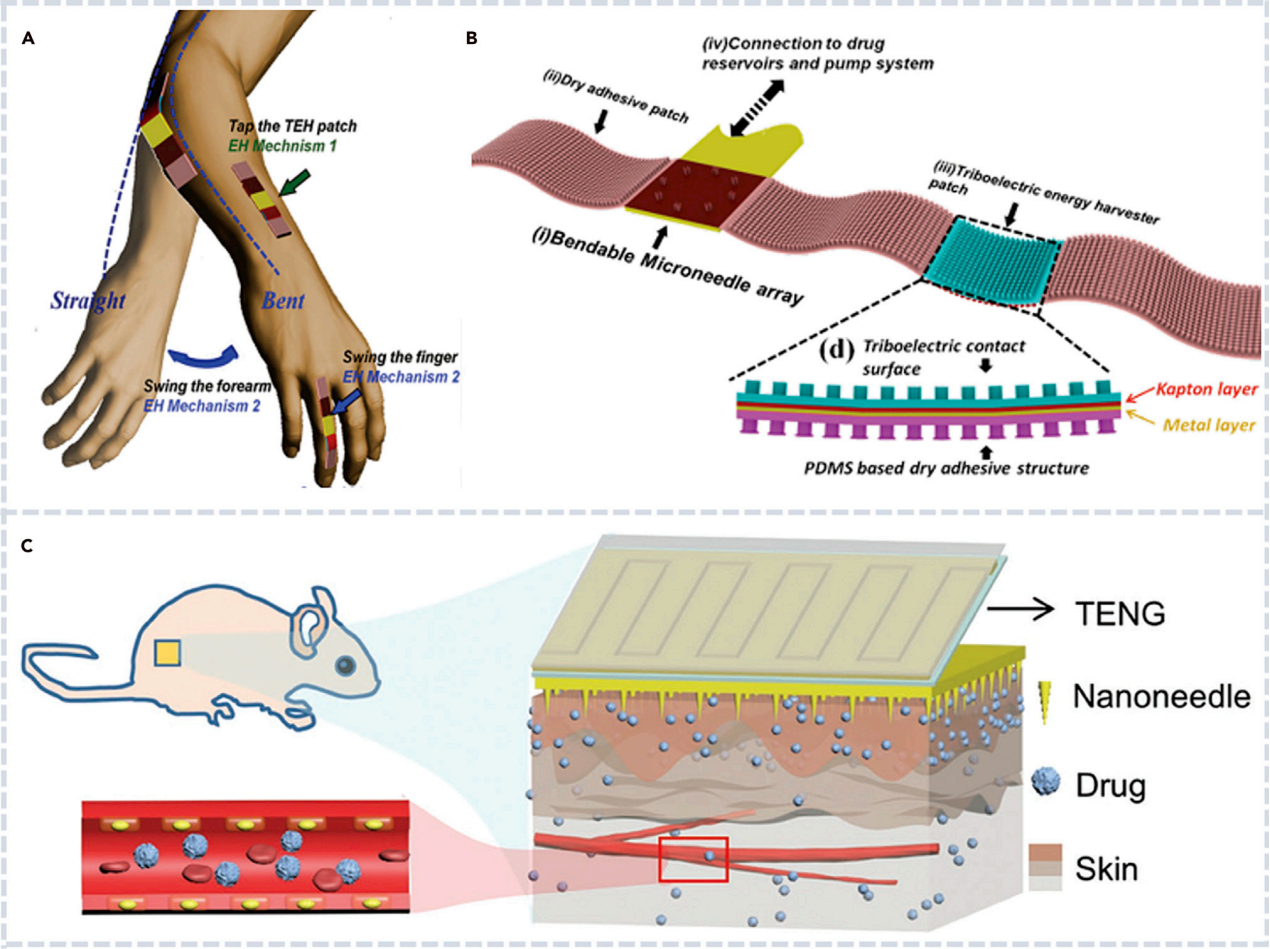
Biocompatible nanogenerators for drug delivery applications (A) Schematic of flexible microneedle skin patches attached on an arm, elbow, and knuckle. The patch consists of four functional components integrated on a whole PDMS sheet: microneedle patch, dry adhesive patch, TEH patch, and pump system (Wang et al., 2016a). (B) Detailed structure and functional components of a flexible microneedle skin patch (Wang et al., 2016a). (C) *In vivo* electroporation drug delivery with a biocompatible TENG (Liu et al., 2019).

Drug delivery with precise control is strongly demanded for both fundamental biological research and therapeutic purpose. In recent years, drug delivery under electrical stimulation by biocompatible TENGs is emerging as a promising technology (Liu et al., 2019; Song et al., 2017; Wang et al., 2016a). Lee et al. developed a stretchable and flexible TENG-based device for transdermal drug delivery (Figure 12A). The delivery function had been confirmed by *in vivo* animal experiments, in which the controlled release of drug was achieved by controlling the pressing force (Figure 12B) (Wang et al., 2016a). Later on, Li et al. developed a TENG-driven electroporation system and realized *in vitro* intracellular and *in vivo* drug delivery in a living mice with high efficiency and minimal cell damage (Figure 12C) (Liu et al., 2019). Thanks to the high local electrical field in a limited area of the nanoneedle-cell interface, the device had high delivery efficiency and caused minimal cell damage. The self-powered and wearable biomechanical energy-powered TENG enabled transdermal on-demand delivery of macromolecules into the mouse tissue, with minimal skin irritation and good compliance. Song et al. achieved a TENG-based self-powered implantable drug delivery system for ocular drug delivery. The controlled release of drug was achieved by controlling the pumping flow rate under different rotating speeds of the TENG, which was indeed controlled by human hand motion (Song et al., 2017).

## Conclusion and outlooks

**Figure 13:**
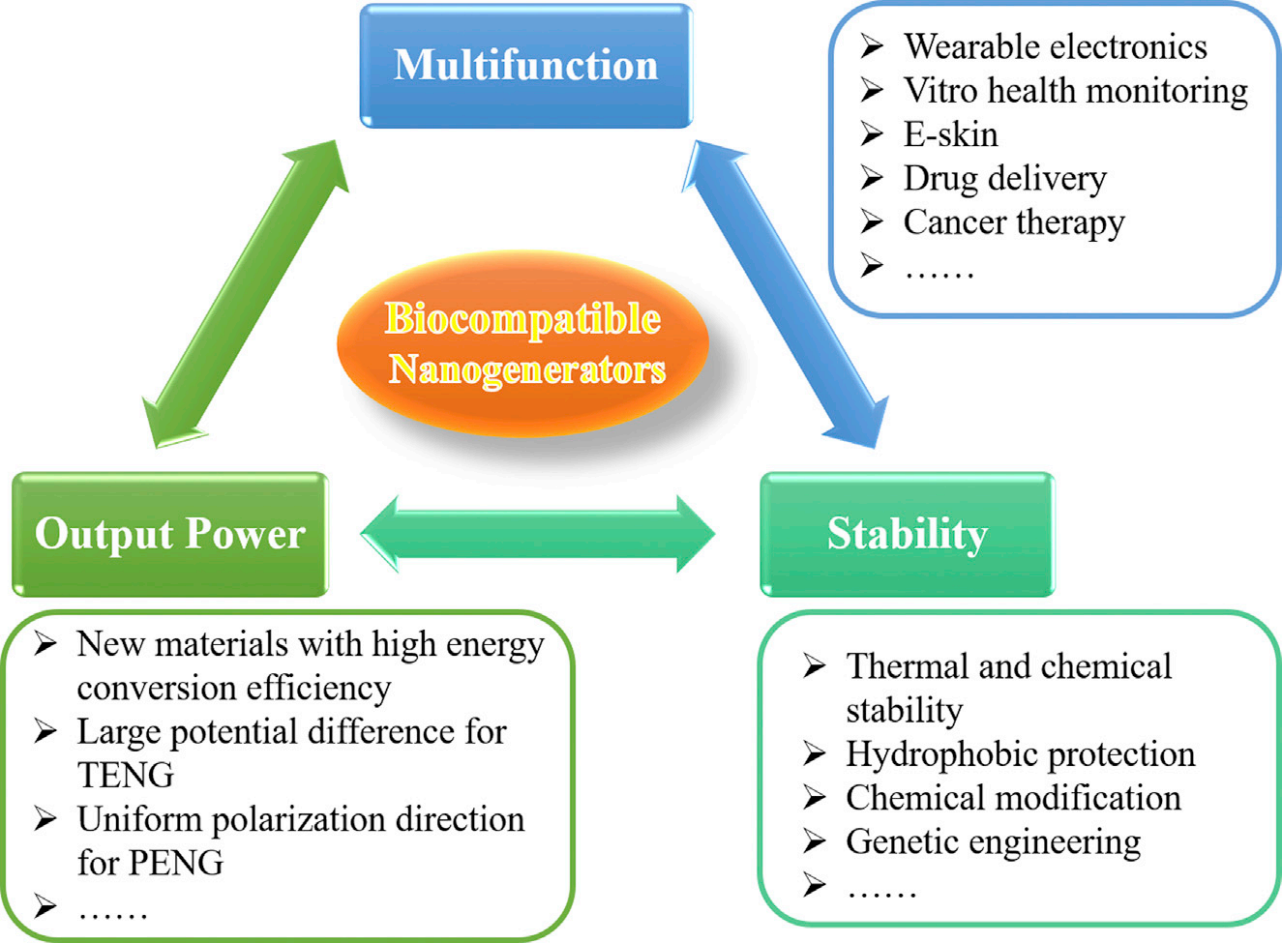
Roadmap about the future of biocompatible nanogenerators

We have reviewed different kinds of biocompatible nanogenerators, such as biocompatible PENGs, biocompatible TENGs, and pyroelectric nanogenerators. Piezoelectric and non-piezoelectric natural and synthetic materials have been used to fabricate biocompatible nanogenerators. These nanogenerators have great implications in various applications due to their material abundance, simple processing method, and environment friendliness. It is worth to mention that nanogenerators can convert human motion energy into electricity, which is important for the development of wearable electronics, *in vitro* health monitoring, and e-skin applications. In addition, the high local electrical field in the limited area produced by biocompatible nanogenerators enables applications for drug delivery and cancer therapy. Even though biocompatible nanogenerators have been widely studied, there are still some challenges in practical applications, especially for implanting applications (Figure 13).

Firstly, the output power of existing biocompatible PENGs is still low. The mechanical energy level of biomotion itself is usually very low, which makes the energy harvesting extremely difficult. It requires the material used in the nanogenerator to be both biocompatible and very efficient in energy conversion. Piezoelectric biomaterials are emerging materials for PENGs, and good piezoelectric properties are desired. However, the adjacent domains in piezoelectric biomaterials, such peptide microstructures/nanostructures, can have random polarization directions and output from these domains can cancel each other out. Complex hydrogen bonding structures make it extremely difficult to unify polarization of biomaterials through a post-growth poling process with a high electric field. So, it is urgently needed to develop growth methods to achieve piezoelectric biomaterials with uniform polarization directions.

Secondly, the stability of biocompatible nanogenerators is a key factor in the commercial applications. Thermal and chemical stability of functional biomaterials in these nanogenerators should be taken into consideration. Till now, there is a lack of research on hydrophobic protection of soluble bio-materials. In addition, crystalline structural instability was observed at high humidity and high temperature. Changes of crystal structure will lead to huge change in their properties which will severely affect the performance of biocompatible nanogenerators. Biomaterials can be tailored by chemical modification, genetic engineering, and other methods to enhance their stability and expand their capability.

Despite such challenges, the development of biocompatible nanogenerators is gaining more and more attention at a fast pace. The emerging new materials and advanced manufacturing methods greatly advances biocompatible nanogenerators and enable new applications in broader fields.
